# Maternal regulation of translation capacity across generations

**DOI:** 10.1371/journal.pbio.3003727

**Published:** 2026-04-10

**Authors:** Elif Sarinay Cenik

**Affiliations:** Molecular Biosciences Department, University of Texas at Austin, Austin, Texas, United States of America

## Abstract

Animals must continually adjust their proteome to match environmental conditions, but it is unclear whether the proteome can be propagated from parent to offspring. This Primer explores a recent study in PLOS Biology showing that maternal nutrient sensing regulates ribosome deposition into embryos, thereby modulating early growth and development.

## Teaser

How animals allocate resources between growth, maintenance, and reproduction is a fundamental question. In this issue of *PLOS Biology*, Pradhan and colleagues show that maternal nutrient sensing regulates ribosome deposition into embryos, shaping the early growth dynamics in the next generation.

Ribosomes are among the most abundant macromolecular complexes in the cell, and in bacteria their abundance is tightly coupled to growth rate. Regulatory circuits couple nutrient availability to ribosome synthesis so that cells increase translational capacity when biosynthetic demand increases [1]. In multicellular animals, however, this relationship is embedded in a more complex physiological context. Growth must be coordinated across tissues and developmental stages, and organisms must balance investment in their own somatic maintenance with the resources allocated to reproduction and offspring fitness.

The nematode *Caenorhabditis elegans* provides a powerful system for studying these questions. This animal develops rapidly, produces large numbers of offspring, and has an invariant somatic lineage [2], making it possible to examine how maternal physiology influences developmental outcomes in the next generation. Early development is particularly informative. Ribosome biogenesis is complex and energetically costly [3], yet embryogenesis in *C. elegans* relies on maternally deposited ribosomes [4]. Zygotically produced ribosomes begin to enter actively translating pools only after hatching, when L1 larvae start feeding and transition into rapid post-embryonic growth [5]. Comparable developmental partitioning of ribosome populations has been observed in vertebrates such as zebrafish, where maternal and somatic ribosomal RNA variants are expressed at different stages and hybrid ribosomes containing maternal and somatic subunits can form during development [5,6]. Together, these findings indicate that embryos begin life with a substantial maternal investment in translational machinery.

**Fig 1:**
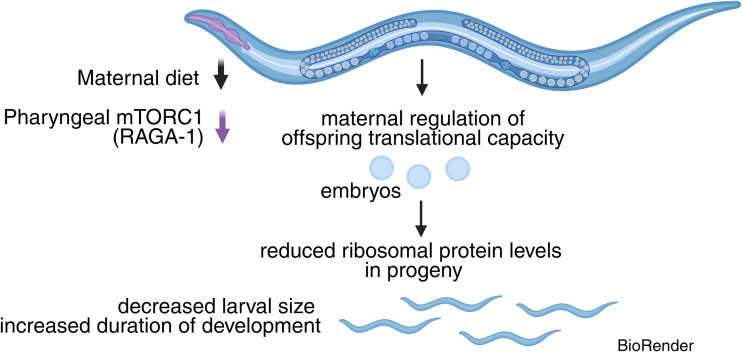
Maternal nutrient sensing regulates ribosome provisioning to the next generation. Maternal dietary restriction and reduced mTORC1 signaling in the pharynx through the Rag GTPase RAGA-1 regulate the amount of translational capacity deposited into embryos. As a result, progeny of dietarily restricted mothers inherit reduced levels of ribosomal proteins at hatching. Although embryogenesis proceeds normally, larvae begin life with lower translational capacity, leading to smaller size and slower early growth until ribosome levels recover during larval development. The diagram was generated in BioRender*. Cole,* E. *(2026)* *https://BioRender.com/zrk057d*.

In this issue of *PLOS Biology*, Pradhan and colleagues asked whether this maternal investment is fixed or instead responds to the physiological state of the mother [7]. Using quantitative proteomics and live imaging, they show that dietary restriction profoundly alters the maternal proteome but that most of these changes are largely reset in the next generation. Ribosomal proteins emerge as a striking exception to this. Progeny of dietarily restricted mothers hatch with lower ribosomal protein levels, smaller body size, and slower early growth under conditions where nutrients are not limited (Fig 1).

Interestingly, reduced ribosome provisioning does not prevent development. Embryos still complete embryogenesis and hatch successfully, and ribosomal protein levels recover during the first larval stage. Because early embryos rely entirely on maternally supplied ribosomes, one might expect reduced provisioning to compromise embryogenesis itself. Instead, the phenotype becomes apparent only after hatching, when larvae enter a phase of rapid biosynthetic demand. These observations suggest that maternal ribosome deposition provides embryos with sufficient translational capacity to complete embryogenesis, while still allowing variation in the ribosome pool that shapes how efficiently larvae initiate post-embryonic growth. Thus, there is a separation between successful embryogenesis and reduced post-embryonic growth. Consistent with this interpretation, the growth disadvantage of progeny from dietarily restricted mothers is attenuated when mTORC1 signaling is inhibited in the offspring, conditions in which maximal protein synthesis capacity is less limiting.

The study further suggests that ribosome provisioning reflects an active maternal allocation decision rather than a passive consequence of reduced maternal growth. Direct experimental reduction of maternal ribosomal protein loading is sufficient to reproduce the early-growth delay, demonstrating that ribosome levels themselves can determine early-growth dynamics. Moreover, maternal inhibition of mTORC1 signaling, either globally or specifically in the pharynx through depletion of the Rag GTPase RAGA-1, similarly reduces ribosomal protein levels in progeny (Fig 1).

A particularly interesting aspect of the study is the tissue specificity of this effect. Depletion of RAGA-1 in the maternal pharynx reduces ribosomal protein levels in the next generation, whereas depletion in the epidermis does not, despite strongly impairing maternal growth. This finding suggests that progeny ribosome provisioning does not simply scale with maternal body size or overall biomass production. Instead, specific tissues may act as physiological sensors that transmit information about environmental conditions to the germline.

The pharynx, which controls food intake and directly senses nutrient availability, may therefore play a key role in communicating dietary conditions to reproductive tissues [8]. By contrast, the epidermis, although important for systemic growth control [9,10], likely reflects the organism’s growth state rather than nutrient intake itself. This distinction raises an interesting possibility: TOR signaling may propagate differently across tissues, with certain organs functioning as primary nutrient sensors whose signals ultimately influence reproductive investment.

A future direction will be to determine how signals from maternal nutrient-sensing tissues are transmitted to the germline. The pharynx could influence ribosome provisioning indirectly by changing feeding behavior and systemic metabolism, but the tissue specificity of the phenotype raises the strong possibility of a more active soma-to-germline signaling mechanism. Why pharyngeal, but not epidermal, inhibition of RAGA-1 alters offspring ribosome content remains especially intriguing, as it suggests that some organs may function as prioritized nutrient sensors whose outputs shape reproductive investment. Understanding the signaling communication routes will help clarify how maternal physiology is translated into changes in embryonic provisioning. Another important future direction will be to determine whether the germline enforces a buffered minimum ribosome load sufficient for embryogenesis, while allowing additional variation that tunes the capacity for rapid post-embryonic growth.

These findings suggest a model in which maternal nutrient sensing regulates the intergenerational allocation of translational capacity. Rather than passively inheriting the maternal proteome, offspring receive a ribosome load allocated by the mother to reflect her nutritional environment. In this view, maternal ribosome provisioning is buffered enough to support embryogenesis yet tuned to influence how rapidly offspring can exploit favorable growth conditions after hatching.
